# Prevalence and Molecular Mechanisms of Carbapenem Resistance among Gram-Negative Bacilli in Three Hospitals of Northern Lebanon

**DOI:** 10.3390/antibiotics11101295

**Published:** 2022-09-22

**Authors:** Mariam Rima, Saoussen Oueslati, Laura Dabos, Dina Daaboul, Hassan Mallat, Elie Bou Raad, Marcel Achkar, Osman Mawlawi, Sandrine Bernabeu, Rémy A. Bonnin, Delphine Girlich, Marwan Osman, Monzer Hamze, Thierry Naas

**Affiliations:** 1 Team ReSIST, INSERM U1184, School of Medicine, Université Paris-Saclay, LabEx LERMIT, 94270 Le Kremlin-Bicêtre, France; 2 Laboratoire Microbiologie Santé et Environnement (LMSE), Doctoral School of Sciences and Technology, Faculty of Public Health, Lebanese University, Tripoli 1300, Lebanon; 3 Bacteriology-Hygiene Unit, Bicêtre Hospital, APHP Paris-Saclay, 94270 Le Kremlin-Bicêtre, France; 4 Clinical Laboratory, El Youssef Hospital Center, Halba 1302, Lebanon; 5 Clinical Laboratory, Nini Hospital, Tripoli 1300, Lebanon; 6 Clinical Laboratory, Tripoli Governmental Hospital, Tripoli 1300, Lebanon; 7 French National Reference Center for Antibiotic Resistance: Carbapenemase-Producing Enterobacterales, 94270 Le Kremlin-Bicêtre, France; 8 Cornell Atkinson Center for Sustainability, Cornell University, Ithaca, NY 14853, USA; 9 Department of Public and Ecosystem Health, College of Veterinary Medicine, Cornell University, Ithaca, NY 14853, USA

**Keywords:** antimicrobial resistance, carbapenem, carbapenemase, Gram-negative bacilli

## Abstract

Carbapenem resistance (CR) is an emerging health issue. Epidemiological surveys on carbapenem-resistant Gram-negative bacilli (CR-GNB) in Lebanon remain scarce. In this study, we determined the prevalence of CR-GNB isolated between 2015 to 2019 in three hospitals in northern Lebanon: 311 CR-*Enterobacterales* (out of 11210; 2.8%), 155 CR-*Pseudomonas* (out of 1034; 15%) and 106 CR- *Acinetobacter* (out of 184; 57.6%) were identified. CR mechanisms were determined for 146 randomly chosen isolates: the Carba NP test revealed an enzymatic resistance to carbapenems in 109 isolates (out of 146, 74.7%). Produced carbapenemases were evaluated by the NG-Test Carba5, NG-Test OXA-23 immunochromatographic assays and PCR. Carbapenemase-producing (CP) *Enterobacterales* expressed *bla*_OXA-48_-like, *bla*_NDM_-like and *bla*_VIM_-like genes and CP-*Pseudomonas* expressed *bla*_IMP_-like and *bla*_VIM_-like genes, whereas CP-*Acinetobacter* expressed *bla*_OXA-23_-like genes. The NG-Test Carba5 results were confirmed by PCR sequencing and revealed several variants, such as NDM-19, VIM-62 and OXA-162, never described so far in Lebanon. Isolates with discordant results were sequenced by WGS and highlighted novel variants of the natural oxacillinases of *Pseudomonas aeruginosa*: *bla*_OXA-50_-like genes. Their role in carbapenem resistance should be further studied. Overall, our findings highlight an alarming situation and encourage health care centers to establish performant registration systems that could help in limiting resistance spread.

## 1. Introduction

Antibiotic-resistant bacteria emergence is increasing drastically worldwide, thus limiting the efficacy of antibiotics. Their overuse or misuse, and the low rate of new drug development in the pharmaceutical industry, make the problem worse. Gram-negative multidrug-resistant bacterial infections remain one of the most dangerous public health threats, and carbapenems constitute the last-resort class of antibiotics for such infections because of their stability against β-lactamases and their broad spectrum of action. For these purposes, resistance to carbapenem should never be underestimated. Mechanisms of this resistance are emerging by several ways, including: (i) impermeability, (ii) efflux, (iii) alteration of the target site and (iv) enzymatic inactivation. The production of enzymes remains one of the leading causes for carbapenem and other β-lactam inactivation, hence the importance of studying the various emerging β-lactamases [1]. Over-production of efflux pumps and the production of AmpC-type β-lactamase accompanied by the reduction of porin expression were also reported to play a major role in carbapenem-resistant strains [2]. According to Ambler, β-lactamases are classified in four groups based on their amino acid sequence differences. Classes A, C and D are serine-based enzymes with a serine residue in their active site, while in class B, we find metallo-β-lactamases (MBL) that require a divalent cation, usually Zn^2+^, for their activity [3]. Carbapenemases belong to class A serine β-lactamases, class B MBLs and class D oxacillinases of Ambler’s classification, with KPC (*Klebsiella pneumoniae* carbapenemase), NDM (New Delhi metallo-β-lactamase), VIM (Verona Integron-encoded MBL), IMP and OXA-48-like (Oxacillinases) being the major carbapenemases worldwide, and classes B and D being dominant in Lebanon [4].

Despite the lack of official national data on antimicrobial resistance (AMR) in Lebanon, each hospital generates its own annual data of antibiotic sensitivity [5]. Data generated by hospitals each year and several publications describing the epidemiology in north Lebanon show that this region follows the same global trend of increasing AMR [5]. The rate of isolates exhibiting reduced susceptibility or resistance to ertapenem increased from 0.4% in 2008–2010 to 0.9% in 2011 and 1.6% in 2012, according to a study conducted by Beyrouthi et al. [6]. This rise was associated with the emergence of the carbapenemase OXA-48. Other nationwide studies underscored the evolution of the prevalence of carbapenem resistance in Lebanon. According to Chamoun et al., *Enterobacterales* had resistance values ranging from 0.7 to 2% over 2011, 2012 and 2013 [7]. High levels of resistance were also noted in *Acinetobacter* spp. and *Pseudomonas* spp. resistance, which exceeded 20% [7]. Other data collected in 2015 and 2016 from three hospitals revealed carbapenem resistance in 3% of *Enterobacterales*, 30% of *Pseudomonas* spp. and 88% of *Acinetobacter* spp. [5]. Carbapenemases encountered in *Enterobacterales* in Lebanon are mostly OXA-48-like enzymes [6]. Carbapenem resistance in *Pseudomonas aeruginosa* is mostly related to non-enzymatic mechanisms, especially those involving mutations in the OprD, the porin responsible for carbapenem uptake, but VIM-2, IMP-1, IMP-2 and IMP-15 carbapenemases have also been described [8]. The prevalence of OXA-23 of *Acinetobacter baumannii* is considerably high in Lebanon [9]. For instance, 91.3% of carbapenem-resistant isolates collected in a study conducted by Dahdouh et al. harbored OXA-23 [10]. Carbapenemases circulate in both environmental and clinical bacterial strains. Carbapenemases present in environmental strains isolated from north Lebanon had OXA-48, OXA-244 and NDM-1 noted mainly in *Enterobacteriaceae*, VIM-2 in *Pseudomonas* and OXA-23, OXA-24, OXA-58, OXA-72 and OXA-143 in *Acinetobacter* [11]. In the following study, we aimed at depicting the type of resistance that circulates in northern Lebanon and pinpointing the genes responsible for enzymatic resistance to carbapenems. The prevalence data of carbapenem resistance collected from three hospitals in northern Lebanon—Nini hospital, El Youssef Hospital Center, and Tripoli Governmental Hospital—from clinical samples between 2015 and 2019, were elaborated. Molecular characterization was carried out on 146 randomly selected Gram-negative bacterial isolates with carbapenem resistance. These include 72 *Enterobacterales*, 29 *P. aeruginosa* and 45 *A. baumannii* isolates.

## 2. Results

### 2.1. Carbapenem Resistance Levels among Gram-Negative Bacteria

In 2015, the prevalence of carbapenem-resistant *Enterobacterales* was 1.4%; this prevalence increased to reach 3.3% in 2019, with a peak noted in 2017 (4.5%). The total prevalence among the 5 years is 2.7%. In *Pseudomonas* sp. Isolates, an increasing trend from 8.1% in 2015 to 27.3% in 2019 was noted. In *Acinetobacter* sp. isolates, the annual prevalence varied by year, with the highest percentage reached in 2016 (83%) and the lowest noted in 2017 (38.7%) (Table 1).

**Figure 1:**
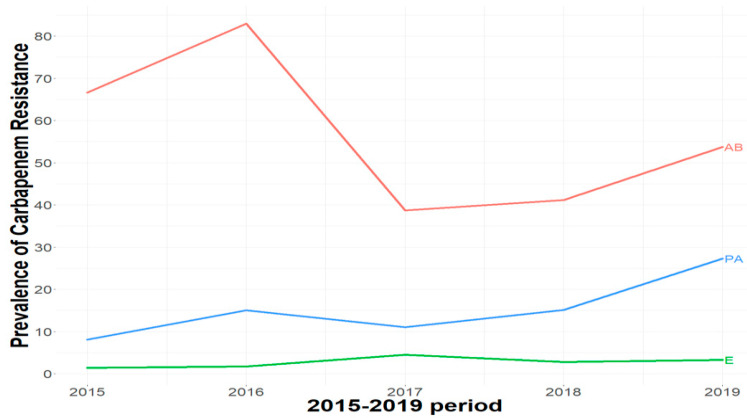
The evolution of carbapenem resistance among *Enterobacterales* (E), *P. aeruginosa* (PA) and *A. baumannii* (AB) isolates in northern Lebanon from 2015 to 2019.

The evolution of carbapenem resistance is presented in Figure 1. No significant temporal trends of carbapenem resistance were detected by the Mann–Kendall test and Sen’s slope among *Enterobacterales* (Z = 1.22, Sen’s = 0.005, *p*-value = 0.221), *P. aeruginosa* (Z = 1.71, Sen’s = 0.041, *p*-value = 0.086) and *A. baumannii* (Z = −0.24, Sen’s = −0.059, *p*-value = 0.807) over the study period (2015–2019). In total, 146 isolates were randomly picked for molecular characterization, yet were representative of the global prevalence in each species. These included 51 *Escherichia coli*, 11 *Klebsiella pneumoniae*, one *Klebsiella variicola*, seven *Enterobacter* spp., two *Citrobacter freundii*, 29 *P. aeruginosa* and 45 *A. baumannii* isolates. 

**Figure 2:**
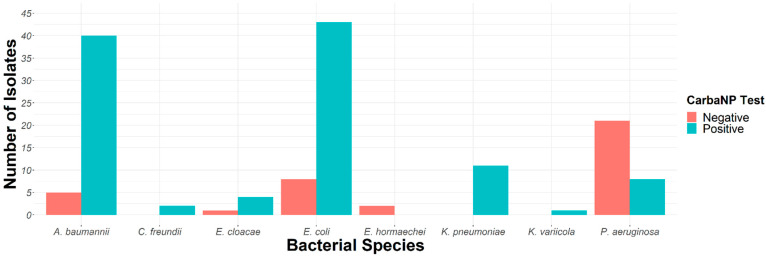
Carba NP test (*Enterobacterales* and *P. aeruginosa*) and CarbAcineto (*A. baumannii*) test results.

All *K. pneumoniae* and *C. freundii* isolates and the majority of *E. coli* (84.3%) and *E. cloacae* (80%) isolates were positive with the Carba NP test, thus suggesting the presence of a carbapenemase. However, all *Enterobacter hormaechei* isolates were negative for the Carba NP test, suggesting a non-enzymatic resistance mechanism to carbapenems (Figure 2). In total, 84.7% of *Enterobacterales* displayed carbapenem-hydrolyzing activity. Similarly, the majority of *A. baumannii* (88.9%) were positive using the CarbAcineto NP test, suggesting the presence of a carbapenem-hydrolyzing enzyme. In contrast, only 27.6% (8/29) of the *P. aeruginosa* isolates had a positive Carba NP test (Figure 2). Altogether, carbapenem resistance in *Enterobacterales* and *A. baumannii* are linked to enzymatic hydrolysis, while in *Pseudomonas,* non-enzymatic mechanisms are most likely the origin.

### 2.2. Carbapenemase Characterization

The Carba NP test revealed isolates with imipenem hydrolytic activity. In order to identify the type of carbapenemase likely at the origin of carbapenem hydrolysis, lateral flow immunoassay (LFIA) and NG-Test Carba5 (NG-Biotech, Guipry, France) were used for *Enterobacterales* and *P. aeruginosa* and the NG-Test OXA-23 (NG-Biotech) for *A. baumannii.*

*K. pneumoniae* and *C. freundii* isolates expressed OXA-48-like carbapenemases, while *E. cloacae* expressed OXA-48, VIM and/or NDM. *E. coli* isolates were positive for two enzymes: OXA-48 and NDM. In *Pseudomonas*, only class B enzymes were detected, and in *A. baumannii*, the NG-Test Carba5 was negative, and the OXA-23-specific LFIA and the NG-test OXA-23 were positive for 39 out of 45 *A. baumannii* isolates. These results were confirmed by PCR sequencing. All Altogether, NG-Test Carba 5 allowed the detection of carbapenemases in strains with enzymatic resistance, while NG-test OXA-23 and PCR allowed the detection of OXA-23 in *Acinetobacter*. The other isolates, positive for Carba NP, but negative using LFIA or PCR, consist of three *K. pneumoniae*: one *K. variicola*, three *P. aeruginosa* and two *A. baumannii*. They were subjected to whole-genome sequencing.

Sanger sequencing of the entire carbapenemase gene revealed the presence of genes coding for OXA-48, OXA181 and NDM-5 in *K. pneumoniae*; OXA-48, VIM-1, VIM-4 and NDM-1 in *E. cloacae*; OXA-48 in *C. freundii*; OXA-48, OXA-162, NDM-5 and NDM-19 in *E. coli*; IMP-15, VIM-2 and VIM-62 in *P. aeruginosa*; and OXA-23 in *A. baumannii* (Table 2). NDM-19, VIM-62 and OXA-162 have never been described so far in Lebanon.

### 2.3. Whole-Genome Sequencing of Discrepant Results

Nine Isolates (three *K. pneumoniae*, one *K. variivola*, three *P. aeruginosa* and two *A. baumannii*) with positive CarbaNP test results, but negative LFIA results, were further characterized using Illumina WGS and subsequent analysis using software available at the center of genomic epidemiology. In two out of three sequenced *K. pneumoniae*, no carbapenemase was detected: the first one had *bla*_CTX-M-15_, *bla*_SHV-1_ and *bla*_OXA-1_ genes and the other one had *bla*_CTX-M-15_, *bla*_SHV-81-like_ (99.8% nucleotide sequence identity) and *bla*_TEM-1B_ genes, respectively. The *bla*_NDM-1_ gene was identified in the third sequenced *K. pneumoniae*, as well as in *K. variicola*. Three *P. aeruginosa* isolates underwent WGS. Novel variants of the chromosomally encoded genes, *bla*_OXA-50_-like and *bla*_AmpC-PAO_-like, were found; for *P. aeruginosa* 1, *bla*_OXA-50_-like mutant (T16A, K112E) and *bla*_PDC-11_ were found, whereas *bla*_OXA-50_-like (R49C, A133G, A181T) and *bla*_PDC-45_ were noted for the second one, as well as *bla*_OXA-50_-like (R167H, D109E) and *bla*_PDC-1_-like for *P. aeruginosa* 3 (Table 3). The WGS of the *A. baumannii* isolate revealed the presence of the natural and chromosome-encoded *bla*_OXA-64_ and *bla*_ADC-26_ genes, together with a *bla*_OXA-72_ gene encoding an OXA-40-like acquired carbapenem-hydrolyzing oxacillinase, and, for the last strain of *A. baumanni*i, OXA-23, OXA-66 and ADC-73 were found. Furthermore, MLST results revealed that the three *K. pneumoniae* isolates belonged to ST-15, ST-37 and ST-48, the *K. variicola* belonged to ST-3195, the two *P. aeruginosa* isolates belonged to ST-357, ST-893 and ST-277 and the *A. baumannii* belonged to ST-229 and ST-1841 (Table 3).

## 3. Discussion

Despite the efforts put into basic research concerning antimicrobial resistance over the last years in Lebanon, easily accessible epidemiological data remainsomehow scarce due to a lack of or low-performing informatic systems in hospitals and healthcare systems. In the present study, we showed high, yet rising, rates of carbapenem resistance in Gram-negatives isolated over a 5-year period in three hospitals in the northern part of Lebanon.

Several previously published studies describe the antimicrobial susceptibility patterns based on records retrieved from different hospitals all over Lebanon. For example, between 2011 and 2013, resistance to imipenem was 0.7% in *E. coli* and 2% in *Klebsiella* [7]. According to Moghnieh et al., the mean resistance of *Enterobacterales* to imipenem slightly increased in 2016 and accounted for 2% [5]. The carbapenem resistance in *Enterobacterales* even reached alarming rates of 6.6% in 2017 in northern Lebanon [12]. Our results are in line with these previous studies.

As previously reported, carbapenem resistance in *Pseudomonas* spp. was as high as 27% in 2011–2013 and 30% in 2016 [5,7]; our results confirm these data, with an average level of 27.2% in 2019. In *Acinetobacter* spp., high antimicrobial resistance rates have been described in the period from 2011 to 2013 in 16 different Lebanese hospitals, with a imipenem resistance rate varying from 15 to 49% over this period [7]. In our study, 2.8% of *Enterobacterales*, 15% of *P. aeruginosa* and 57.6% of *A. baumannii* are resistant to carbapenem. Those percentages were lower in previous years, except for *Acinetobacter* spp., which showed continuously high resistance rates to most antimicrobials [7]. Careful analysis of our data did not reveal a statistically significant increasing trend of carbapenem resistance in the city of Tripoli, northern Lebanon. The issue of AMR is complicated in low and middle-income (LMIC) countries, such as Lebanon, where many of the factors leading to the emergence and spread of MDR isolates, especially carbapenemase-producing isolates, remain difficult to control.

In the present study, 146 carbapenem-resistant isolates were randomly chosen among the most prevalent species encountered in the clinical settings and presenting reduced susceptibility to ertapenem and/or imipenem. The mechanism sustaining the carbapenem resistance was investigated for 72 *Enterobacterales*, 29 *P. aeruginosa* and 45 *A. baumannii* collected between the years 2015 and 2019. 

Initial biochemical screening using the Carba NP test or CarbaAcineto NP test revealed 109/146 isolates (74.7%) had carbapenem hydrolytic activity. The remaining 37 isolates (25.4 %) had most likely non-enzymatic resistance mechanisms to carbapenems, as revealed by contact growth to a moxalactam antibiotic disc-on-disc diffusion routine antibiogram. Indeed, moxalactam, a ß-lactam belonging to the cephamycins, is rarely hydrolyzed by ß-lactamases, and thus constitute a good marker for impermeability [13]. The most prevalent resistance mechanism in *Enterobacterales* and *A. baumannii* was the production of carbapenemases, unlike *P. aeruginosa,* for which non-enzymatic resistance mechanisms, such as loss of porin D2 and/or the production of a β-lactamase lacking significant imipenem-hydrolyzing properties, associated with permeability problems (loss of porin or hyper expression of the pumps efflux), represented 75%. Isolates with a positive Carba NP test were further studied using LFIAs detecting the five main carbapenemases (KPC, VIM, NDM, IMP and OXA; NG-Test Carba5) and OXA-23-like enzymes (NG-Test OXA-23) from a bacterial colony [14]. These results were subsequently confirmed using the PCR/sequencing approach. 

Our results were in line with previous data that showed that OXA-48-like-producing *Klebsiella* spp. isolates are present in Lebanon [15], but we also evidenced NDM-1 and NDM-5-producing *K. pneumoniae*, described for the first time in Lebanon in 2012 and 2018, respectively [16,17]. In *Enterobacter* spp., OXA-48, VIM-1, VIM-4 and NDM-1 were described, which have already been described in Lebanon [17,18,19]. As in our study, the *E. coli*-producing OXA-48, OXA-162, OXA-181, OXA-244 and NDM-5, have already been described [17,19], but our study reports OXA-162 and NDM-19 for the first time. The OXA-162 carbapenemase was initially reported in Greece in 2015, which is geographically close to Lebanon [20].

In *Pseudomonas*, a novel VIM-2-variant, VIM-62, has never been described to date in Lebanon. As NG-Test Carba5 does not detect OXA-23, which is very common among *Acinetobacter* spp., a companion assay, the NG-Test OXA-23 was performed, and revealed the presence of OXA-23 in all but one *A. baumannii* isolate. In a previous study, OXA-23 was identified as the main carbapenemase in *A. baumannii* in Lebanon, as well as the Mediterranean region [4], which is still the case in our study [21]. For isolates presenting discrepant results (four *Klebsiella* spp., three *P. aeruginosa* and two *A. baumannii)* with positive Carba NP test with negative LFIA results, WGS was carried out. For two *K. pneumoniae* isolates, no carbapenemase gene was found, but the presence of CTX-M-15 ESBLs with disrupted porin-encoding genes are likely at the origin of reduced carbapenem susceptibility, but do not explain the positive Carba NP test, suggesting a false positive test. In one *K. pneumoniae* and one *K. variicola* isolate, WGS revealed the presence of the carbapenemase *bla*_NDM-1_ gene, which was suggested by a faint NG-Test Carba5 signal for NDM and is compatible with the positive Carba NP test. Re-testing of these isolates with the NG-Test Carba5 revealed reproducibly a slight band for NDM, suggesting low expression of NDM1 in these isolates. 

For the *P. aeruginosa* isolates with positive Carba NP tests, negative NG-Test Carba5 and negative for SPM, GES and GIM PCRs, the WGS results revealed the presence of single-point mutant derivatives of the naturally and chromosomally encoded OXA-50 and PDC. These mutations are new, and it is difficult to speculate whether these mutations may contribute to an increased carbapenem hydrolysis, as shown for ACT-28 [22]. Further characterization is necessary to determine the precise role of these variants in the carbapenem-resistant profile. OXA-50 has been initially described as a weak carbapenem-hydrolyzing enzyme [23], and hyperproduction of AMPC may also lead to decreased carbapenem susceptibility. 

The WGS results for the *A*. *baumannii* isolate with a positive Carba NP test, negative NG-Test OXA-23 and OXA-23-specific PCR revealed the presence of the natural and chromosome-encoded OXA-64, without an IS*Aba1* inserted upstream, suggesting a low-level-expressed enzyme and an acquired carbapenemase OXA-72 belonging to the OXA-40 carbapenem-hydrolyzing oxacillinases of A. *baumannii* [24], and thus not detected using the NG-Test OXA-23 and OXA-23-specific PCR. In addition, WGS results revealed different MLSTs for the nine WGS isolates, suggesting sporadic cases rather than outbreak-related isolates.

Carbapenemases observed in our study belonged to classes B and D of Ambler’s classification; however, for Carba NP-negative isolates, the most likely carbapenem resistance mechanism is the production of an ESBL and/or AMPC with low carbapenem-hydrolyzing activity and impaired outer membrane, such as loss of porin or overexpression of efflux pumps [16].

Our results confirm previous findings and reveal a worrying AMR situation in Lebanon, notably during this critical period [25,26,27,28]. This country, which hosts ~1.5 million refugees, is currently experiencing severe economic and political collapse, resulting in a shortage of medicine, appropriate diagnostic tools, food and other essential necessities [29,30,31,32]. Taken together, there is a paramount need to tackle the AMR challenge at national and global levels. Promoting infection control measures, active screening of AMR carriers and antimicrobial stewardship programs are required in healthcare facilities. Finally, as colistin-resistant GNB are increasingly reported over recent years, colistin susceptibility needs to be determined precisely in routine clinical laboratories in Lebanon [26,33,34,35,36,37].

## 4. Materials and Methods

### 4.1. The Bacterial Isolates and Prevalence

**Figure 3:**
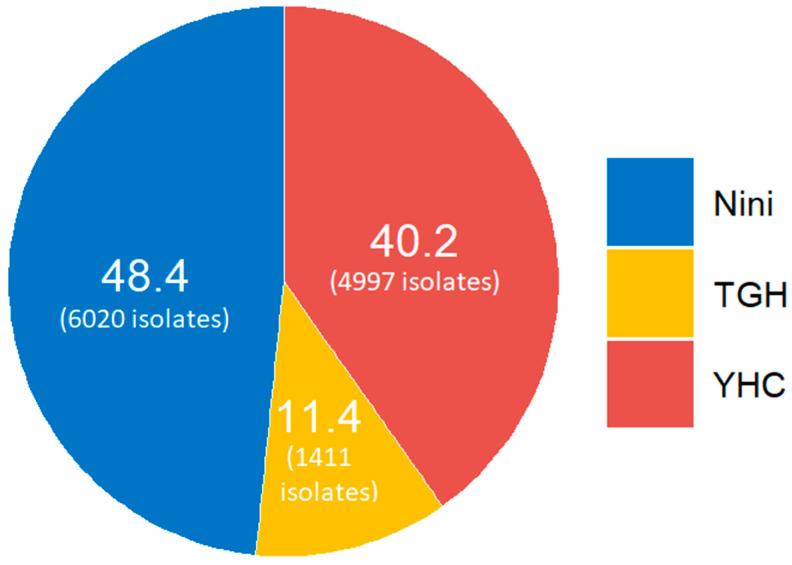
Origin of the isolates (%).

This study is based on records of antimicrobial susceptibility tests performed on 12,428 clinical Gram-negative isolates collected in three hospitals in northern Lebanon—Nini hospital, Al Youssef hospital and Tripoli governmental hospital—between 2015 and 2019, following EUCAST guidelines [38]. The origin of collected isolates is shown in Figure 3. Species were identified by testing through Matrix-Assisted Laser Desorption/Ionization Time-of-Flight Mass Spectrometry (entero-TOF MS). Prevalence percentages were calculated based on antibiotic susceptibility tests made on all the clinical isolates using routine disk diffusion antibiograms. All isolates were from clinical samples, including pus, bronchoalveolar lavages, blood, urine, catheters and sputum.

A total of 146 carbapenem-resistant Gram-negative isolates were picked randomly to study the underlying carbapenem resistance mechanisms. Selected isolates’ number, species, origin of isolation and type of infection are shown in Table 4.

Samples were stored in the biobank of the “Laboratoire de Microbiologie, Santé et Environnement (LMSE)” of the Lebanese University. A total of 41 isolates were picked from Nini Hospital, 27 from Tripoli government hospital and 78 from El Youssef Hospital Center. These included 72 *Enterobacterales*, 29 *P. aeruginosa* and 45 *A. baumannii* isolates. Susceptibility to imipenem and meropenem was further studied by the E-test method and analyzed according to EUCAST clinical breakpoints [38].

### 4.2. Biochemical and Immunoenzymatic Assays for Carbapenemase Detection

The homemade Carba NP test was carried out as previously described [39]. Any change in the color of the solution from red to yellow after two hours incubation at 37 °C was considered positive for imipenem hydrolysis and thus for the presence of a carbapenem-hydrolyzing enzyme. For *A*. *baumannii* isolates, the modified Carba NP test known as the CarbAcineto NP was used [40]. Similarly, a red to yellow color change indicates the presence of a carbapenemase-producing isolate. A control tube with no imipenem was performed for each test.

### 4.3. The Lateral Flow Immunoassays (LFIA)

The NG-Test Carba 5 and the NG-Test OXA-23 (NG Biotech, Guipry, France) were carried out on Carba NP-positive isolates according to the manufacturer’s instructions [14], where one colony grown on Mueller–Hinton agar plates was resuspended in five drops of extraction buffer. 100 μL of this suspension were transferred either to the NG-Test Carba5 cassette or NG-Test OXA-23 (NG Biotech). The results were eye read after 15 min of migration at room temperature.

### 4.4. Molecular Detection of Carbapenemase Genes and Sanger Sequencing

A few colonies of each bacterium to be analyzed were taken from Mueller–Hinton agar plates and resuspended in 100 µL of sterile distilled water. DNA was extracted using the boiling extraction procedure by incubating the tube for 10 min at 95 °C, then for 10 min at −80 °C and 5 min at 95 °C (lysis by thermal shock). Supernatant containing the DNA was collected after centrifugation for 10 min at 10,000 rpm and used for PCR analysis. Carbapenemase genes were sought by PCR using primers specific for *bla*_OXA-48_, *bla*_KPC_, *bla*_NDM_, *bla*_VIM_ and *bla*_IMP_, as previously described [41].

PCR products were analyzed by agarose gel electrophoresis containing ethidium bromide [41]. DNA migrated under a voltage of 120 V, at an amperage of 400 mA, for 30 min. Reading took place under a UV lamp (Ultra-Violet) with an imager, integrating the Vision Capt computer software. Each PCR product positive for the gene tested was purified using Genejet PCR purification Kit^®^ (Thermofisher, Les Ulis, France) according to the manufacturer’s instructions. Each purified PCR product was subsequently sequenced using the Big Dye^TM^ Terminator V1.1 and Applied Biosystem 3130 genetic analyzer and run on an Applied Biosystem 3130 automated sequencer (Applied Biosystems, Les Ulis, France), as previously described [41].

### 4.5. Whole-Genome Sequencing

Genomic DNA was extracted using the PureLinkTM genomic DNA Mini Kit (Thermofischer) following the manufacturer’s instructions. Dual-indexed sequencing libraries were constructed using the NEBNext^®^ library preparation kit and the Multiplex Oligos for Illumina^®^ (NEB, Boston, MA, USA). Libraries were pooled and 100 pM were sequenced on the Illumina Next 500 (2 × 150 bp). Genome assembly was performed using CLC Genomics Workbench v12 (Qiagen, Les Ulis, France). Resistant genes were identified using ResFinder 4.1 [42], and multi locus sequence type (MLST) was determined using MLST V2.0.42 [43].

### 4.6. Nucleotide Sequence Accession Number

The whole genome sequences generated in the study have been submitted to the Genbank nucleotide sequence database, Bioproject PRJNA820555, under the accession numbers of JALHBC000000000, JALHBA000000000, JALHBB000000000, JALHAY000000000, JALHAX000000000, JALHAW000000000 and JALHAZ000000000 (Table 3).

### 4.7. Statistical Analysis

Data were analyzed using R software (R Core team, version 4.1.0; R Studio, version 2022.07.1+554). We used non-parametric statistical tests, the Mann–Kendall test and Sen’s slope to detect monotonous temporal trends of carbapenem resistance among *Enterobacterales*, *P. aeruginosa* and *A. baumannii* over the study period (2015–2019). Figures were illustrated using the ggplot2 R package.

## 5. Conclusions

Our study revealed high rates of carbapenem resistance at the level of three north Lebanese hospitals in *Enterobacterales*, *P*. aeruginosa and *A. baumannii*. Several variants, including NDM-19 and OXA-162 (in *E. coli*), and VIM-62 (in *P. aeruginosa*), have never been described so far in Lebanon, which suggests an ongoing spread of variants of these enzymes. Novel OXA-50-like variants have also been identified, but further characterization is required to determine their precise contribution to the overall carbapenem resistance. In the present study, imipenem-resistant isolates were studied, but as carbapenemase producers may have MICs below the breakpoints, it might well be that our numbers are underestimated. Future work is required, using the EUCAST epidemiological screening cut off for meropenem or ertapenem to increase the potential isolates expressing a carbapenemase. To confirm that the spread of these variants is sporadic, the number of strains analyzed must be increased, in addition, the complete genome of a significant number of strains must be determined.

## Figures and Tables

**Table 1:** Prevalence of carbapenem resistance in *Enterobacterales*, *P. aeruginosa* and *A. baumannii*, from 2015 to 2019, isolated in northern Lebanon, selected based on their resistance to ertapenem according to EUCAST guidelines [10].

Year	*Enterobacterales*		*P. aeruginosa*		*A. baumannii*	
	N	Resistant isolates (%) ^a^	N	Resistant isolates (%) ^a^	N	Resistant isolates (%) ^a^
2015	2081	31 (1.4%)	244	20 (8.1%)	18	12 (66.7%)
2016	2157	37 (1.7%)	193	29 (15%)	47	39 (83%)
2017	1696	78 (4.5%)	172	19 (11%)	31	12 (38.7%)
2018	2125	60 (2.8%)	238	36 (15.1%)	34	14 (41.2%)
2019	3151	105 (3.3%)	187	51 (27.3%)	54	29 (53.7%)
Total	11,210	311 (2.7%)	1034	155 (15%)	184	106 (58%)

^a^ Carbapenem-resistant isolates.

**Table 2:** Carbapenemases identified by Sanger sequencing in different multidrug-resistant GNB and the origin of each isolate.

Bacteria	Number of Isolates	CarbaNP-Positive Isolates	Carbapenemases (Sanger Sequencing)
*K. pneumoniae*	*n* = 11	11 (100%)	OXA-48 (6) OXA-181 (1) NDM-5 (1) (3) ^a^
*K. variicola*	*n* = 1	1 (100%)	(1) ^a^
*E. cloacae*	*n* = 5	4 (80%)	OXA-48 (1) VIM-4 (1) VIM-1 (1) NDM-1 (1)
*E. hormaechei*	*n* = 2	0	-
*C. freundii*	*n* = 2	2 (100%)	OXA-48 (2)
*E. coli*	*n* = 51	43 (84%)	OXA-48 (11) OXA-162 (1) OXA-181 (12) OXA-244 (6) NDM-5 (4) NDM-19 (9)
*P. aeruginosa*	*n* = 29	8 (27%)	VIM-62 (2) VIM-2 (2) IMP-15 (1) (3) ^a^
*A. baumannii*	*n* = 45	33 (73%)	OXA-23-LIKE (31) (2) ^a^

^a^ WGS performed.

**Table 3:** WGS analysis of selected isolates using Resfinder and MLST.

Species	MICs (μg/mL)		Phenotypic Tests		Resfinder		MLST	Accession Numbers
	IMP	MER	Carba NP	NG-Test Carba5	ß-Lactamases Genes	Other Resistant Genes		
*K. pneumoniae* 1	0.19	0.032	+	-	*bla*_CTX-M-15_, *bla*_SHV-1_, *bla*_OXA-1_	*aac(6′)-Ib-cr, aac(3)-Iia, OqxA, OqxB, fosA, tet(A*)	ST-48	JALHBC000000000
*K. pneumoniae* 2	0.38	1.5	+	-	*bla*_CTX-M-15_, *bla*_SHV-81_, *bla*_TEM-1-B_	*sul1, sul2, oqxA, dfrA17, OqxA, OqxB,* *qacE, aadA5, tet(A), fosA*	ST-37	JALHBA000000000
*K. pneumoniae* 3	6	6	+	Faint NDM band	*bla*_NDM-1_, *bla*_CTX-M-15_, *bla*_SHV-106_	*aac(6′)-Ib-cr, msr(E), sul1, OqxA, dfrA12, OqxB, fosA, aadA2, mph(E)*	ST-15	JALHBB000000000
*K. variicola*	1.5	2	+	Faint NDM band	*bla*_NDM-1_, *bla*_SHV-12_, *bla*_LEN-9_, *bla*_OXA-10_, *bla*_OXA-1_	*aac(6′)-Ib-cr, rmtC, rmtH, erm(B), aph(3′)-Ia, sul1, sul2, OqxB, OqxA, dfrA14, floR, qacE, fosA, qacE, tet(B), aadA1, ARR-2, catB3, cmlA1, catA2*	ST-3195	JALHAY000000000
*P. aeruginosa* 1	>32	>32	+	-	*bla*_OXA-50_-like (T16A, K112E), *bla*_PDC-11_	*sul1, crpP, fosA, catB7, aph(6)-Id, aph(3″)-Ib*	ST-357	JALHAX000000000
*P. aeruginosa* 2	>32	>32	+	-	*bla*_OXA-50_-like (R49C, A133G, A181T), *bla*_PDC-45_	*aph(3′)-IIb, fosA, catB7*	ST-893	JALHAW000000000
*P. aeruginosa* 3	>32	>32	+	-	*bla*_OXA-50_-like (R167H, D109E), *bla*_PDC-1_-like	*Sul1, fosA, aph(3′)-IIb, crpP, catB7*	ST-277	Pending
*A. baumannii* 1	>32	>32	+	-	*bla*_OXA-72_, *bla*_OXA-64_, *bla*_ADC-26_	*aac(6′)-lan, aph(6)-Id, aph(3″)-lb, aac(3)-Iia, sul2,* *aac(3)-Iia, tet(B)*	ST-229 *	JALHAZ000000000
*A. baumannii* 2	>32	>32	+	-	*bla*_OXA-23_, *bla*_OXA-66_, *bla*T_EM-1D_, *bla*_ADC-73_	*armA, msr(E), aph(3′)-Ia, sul2, tet(B), aph(6)-Id*	ST-1841-like *	Pending

* using the Pasteur MLST scheme.

**Table 4:** Origin and percentage of tested clinical isolates.

Species	N	Hospital ^a^	Type of Infection	Year
*Klebsiella pneumoniae*	11	Nini (7) YHC (4)	– Vaginal secretions (1) – Sputum (2) – Urine (7) – Nasal secretions (1)	2015–2018
*Klebsiella variicola*	1	YHC (1)	– Urine (1)	2018
*Enterobacter cloacae*	5	Nini (3) TGH (2)	– Catheter (2) – Ear swab (2) – Wound (1)	2017–2018
*Enterobacter hormaechei*	2	Nini (1) TGH (1)	– Pus (1) – Deep abdominal wound (1)	2017
*Citrobacter freundii*	2	YHC (2)	– Pus (2)	2017
*Escherichia coli*	51	Nini (22) YHC (25) TGH (4)	– Urine (34) – Pus (7) – Blood (2) – Nasal secretions (1) – Gastric liquid (4) – Sputum (1) – Wound (1) – Rectal swab (1)	2016–2018
*Pseudomonas aeruginosa*	29	YHC (25) TGH (4)	– Urine (18) – Blood (3) – Pus (5) – Wound (2) – Catheter (1)	2016–2018
*Acinetobacter baumannii*	45	Nini (8) YHC (22) TGH (15)	– Sputum (19) – Catheter (5) – Tracheal aspiration (3) – Cavum (1) – Wound (5) – Urine (1) – Blood (2) – Pleural liquid (1) – Bronchoalveolar lavage (4) – Pus (4)	2016–2019

^a^ Nini: Nini hospital; TGH: Tripoli governmental hospital; YHC: Youssef Hospital Center.

## Data Availability

WGS sequences have been submitted to Genbank database.
